# Prevalence of Antibiotic Tolerance and Risk for Reinfection Among *Escherichia coli* Bloodstream Isolates: A Prospective Cohort Study

**DOI:** 10.1093/cid/ciac281

**Published:** 2022-04-22

**Authors:** Gilad Lazarovits, Orit Gefen, Noga Cahanian, Karen Adler, Ronen Fluss, Irit Levin-Reisman, Irine Ronin, Yair Motro, Jacob Moran-Gilad, Nathalie Q Balaban, Jacob Strahilevitz

**Affiliations:** Faculty of Medicine, Hebrew University of Jerusalem, Jerusalem, Israel; Department of Pediatrics, Hadassah Hebrew University Medical Center, Jerusalem, Israel; Racah Institute of Physics, The Hebrew University of Jerusalem, Jerusalem, Israel; Department of Clinical Microbiology and Infectious Diseases, Hadassah Hebrew University Medical Center, Jerusalem, Israel; Faculty of Medicine, Hebrew University of Jerusalem, Jerusalem, Israel; The Biostatistical and Biomathematical Unit, Gertner Institute for Epidemiology & Health Policy Research, Sheba Medical Center, Ramat Gan, Israel; Racah Institute of Physics, The Hebrew University of Jerusalem, Jerusalem, Israel; Racah Institute of Physics, The Hebrew University of Jerusalem, Jerusalem, Israel; Department of Health Policy and Management, School of Public Health, Ben Gurion University of the Negev, Beer Sheva, Israel; Department of Clinical Microbiology and Infectious Diseases, Hadassah Hebrew University Medical Center, Jerusalem, Israel; Department of Health Policy and Management, School of Public Health, Ben Gurion University of the Negev, Beer Sheva, Israel; Racah Institute of Physics, The Hebrew University of Jerusalem, Jerusalem, Israel; Faculty of Medicine, Hebrew University of Jerusalem, Jerusalem, Israel; Department of Clinical Microbiology and Infectious Diseases, Hadassah Hebrew University Medical Center, Jerusalem, Israel

**Keywords:** antimicrobials, bloodstream infection, tolerance

## Abstract

**Background:**

Tolerance is the ability of bacteria to survive transient exposure to high concentrations of a bactericidal antibiotic without a change in the minimal inhibitory concentration, thereby limiting the efficacy of antimicrobials. The study sought to determine the prevalence of tolerance in a prospective cohort of *E. coli* bloodstream infection and to explore the association of tolerance with reinfection risk.

**Methods:**

Tolerance, determined by the Tolerance Disk Test (TDtest), was tested in a prospective cohort of consecutive patient-unique *E. coli* bloodstream isolates and a collection of strains from patients who had recurrent blood cultures with *E. coli* (cohorts 1 and 2, respectively). Selected isolates were further analyzed using time-dependent killing and typed using whole-genome sequencing. Covariate data were retrieved from electronic medical records. The association between tolerance and reinfection was assessed by the Cox proportional-hazards regression and a Poisson regression models.

**Results:**

In cohort 1, 8/94 isolates (8.5%) were tolerant. Using multivariate analysis, it was determined that the risk for reinfection in the patients with tolerant index bacteremia was significantly higher than for patients with a nontolerant strain, hazard ratio, 3.98 (95% confidence interval, 1.32–12.01). The prevalence of tolerance among cohort 2 was higher than in cohort 1, 6/21(28.6%) vs 8/94 (8.5%), respectively (*P* = .02).

**Conclusions:**

Tolerant *E. coli* are frequently encountered among bloodstream isolates and are associated with an increased risk of reinfection. The TDtest appears to be a practicable approach for tolerance detection and could improve future patient management.

## Teaser

Tolerance may lead to the failure of antibiotic therapy. Applying the Tolerance Disk Test, we found that 8.5% of *Escherichia coli* bloodstream infections were tolerant, tolerant strains were more frequent among patients with recurrent bacteremia, and tolerance was an independent risk for reinfection.

Appropriate antibiotic therapy is a key determinant of favorable patient outcome and is based on the susceptibility of bacteria to selected therapy. Antibiotic resistance directly relates to the concentration of the antibiotic that is required to inhibit bacterial growth. Tolerance, a resistance-independent pathway for bacterial survival under antibiotic exposure, is defined as a transient ability of bacteria to survive under an otherwise bactericidal treatment. Tolerance may also lead to the failure of antibiotic therapy and facilitate the emergence of resistance [1–3]. Current antibiotic treatment decisions are based on standardized antimicrobial susceptibility testing, which does not detect tolerance. Hence, tolerant strains may be misclassified as susceptible, thereby resulting in treatment failure. Indeed, tolerant bacteria have been found among clinical isolates and associated with treatment failure [4–11]. These reports, however, mostly involved few selected strains. We used a prospective cohort of bloodstream infections (BSIs) involving *Escherichia coli* to characterize the epidemiology of tolerance more broadly in a clinical population and define the risk of BSIs among patients with prior tolerant *E. coli* BSIs.

## METHODS

### Study Design

The Hadassah-Hebrew University Medical Center is a tertiary medical center serving the Jerusalem urban area consisting of 2 campuses with a total of 1100 admission beds. All patient-unique clinical blood isolates are prospectively collected at the Clinical Microbiology Laboratory and kept at −80°C. The clinical isolates are routinely identified to the species level using matrix-assisted laser desorption ionization–time of flight mass spectrometry (VITEK MS, bioMérieux, Marcy l’Etoile, France) and undergo antimicrobial susceptibility testing using the VITEK2 platform (bioMérieux).

For this study, we used 2 cohorts. The first consisted of 100 consecutive patient-unique *E. coli* blood isolates recovered between November 2017 and March 2018 (cohort 1). This allowed an accurate determination of the incidence of tolerance. Cohort 2 included isolates from patients who had recurrent positive blood cultures of *E. coli* exhibiting the same susceptibility profile, 2 weeks to 1 year apart, between March 2014 and April 2017. This cohort was used to assess the prevalence of tolerance among patients with repeated *E. coli* BSIs.

This study was reviewed by the institutional review board and a waiver from informed consent was granted.

### Covariates

For each isolate, we extracted demographic data, comorbidities, baseline laboratory, and length of stay following index infection.

### In Vitro Analysis of Tolerance

Study isolates were analyzed for the tolerance phenotype using the TDtest and a subset of isolates were also tested using a time-dependent killing assay.

### Tolerance Disk Test

The Tolerance Disk Test (TDtest) was done as previously described [12] with the following modifications: bacterial inocula of approximately 10^7^ bacteria were grown overnight to stationary phase and adjusted using the McFarland standards as a reference. Suspensions were plated on Mueller Hinton agar plates and 5 µL of antimicrobial solution was pipetted onto blank antimicrobial susceptibility Oxoid disks. The amount of ertapenem used was 0.02 µg, 0.2 µg, and 2 µg and plates were incubated overnight at 37°C (step I). If the inhibition zone radius was <1 cm, the experiment was repeated using 0.4 µg and 4.0 µg. The corresponding amounts of ampicillin were 10 µg, and of ceftriaxone 0.02 µg, 0.2 µg, and 2 µg. In step II, 10 µL of a solution containing 40 g/100 mL glucose and 20 g/100 mL casamino acids mixture (BD Biosciences, San Jose, CA, USA) was added to the antibiotic disk and the plates were reincubated at 37°C for an additional overnight. Strains were classified as “tolerant” if the number of microcolonies appearing in the inhibition zone after step II exceeded 20. All chemicals, unless stated otherwise, were purchased from Sigma-Aldrich Chemical Co.

### Time-dependent Killing Experiments

Overnight cultures were diluted 1:100 in fresh medium containing ertapenem, at approximately 20-fold the corresponding minimal inhibitory concentration and incubated at 37°C with shaking, for a designated time. tbl3a was used as a reference tolerant strain [13] and mgy as a wild-type control [14]. Kill was defined as ≥3-log_10_ colony-forming units per milliliter decrease in bacterial counts [15]. Bacterial survival was determined by the most probable number-counting method.

### Strain Typing

Tolerant isolates underwent DNA extraction using the DNeasy Blood & Tissue kit (Qiagen, Hilden, Germany) according to the manufacturer’s instructions. The genomic libraries were constructed using the Nextera FLEX library preparation kit (Illumina Inc., San Diego, CA, US) according to manufacturer’s instructions, followed by sequencing on an Illumina NovaSeq sequencer.

All sequencing reads passed quality control using FastQC [v0.11.7], with a minimum sequencing depth of 20×, and were identified as *E. coli*, while also checking for contamination, using Kraken2 ([16] with the minikraken2_v1_8GB database). Sequences were subject to trimming and de novo assembly using the shovill pipeline (Seemann T. github. https://github.com/tseemann/shovill [accessed August 2021]) (with Trimmomatic [v0.38], SPADes [v3.14.1], and Pilon [v1.22]). All assembled genomes were in silico sequence typed using the tool mlst (v2.19.0) (Seemann T. github. https://github.com/tseemann/shovill [accessed August 2021]) with the “ecoli” scheme from pubMLST [17]. A core genome single-nucleotide polymorphism (SNP)-calling analysis was performed to evaluate the genetic relatedness, using snippy [v4.6.0] (Seemann T. github. https://github.com/tseemann/shovill [accessed August 2021]) (with default parameters). *E. coli* strain ExPEC XM (accession: GCF_002844685.1) was selected as the reference genome by comparing the genome assemblies to complete genome assemblies using the tool referenceseeker [18]. Core genome SNPs were then determined using snippy-core, and recombination sites were masked using Gubbins [v2.4.1] [19]. A minimum spanning tree was generated (using the MSTreeV2 method) and visualized from the final masked cgSNPs alignment with GrapeTree [v1.5.0] [20].

### Statistical Analysis

We compared the risk of reinfection between the 2 groups: patients who had a tolerant index infection vs those who had a nontolerant strain.

The group of patients with tolerant and nontolerant strain BSIs were compared for infection covariates (potential confounders), using *t* test and Fisher exact test for continuous and categorical variables, respectively. The primary outcome was a repeat BSI or urinary tract infection (UTI) within 90 days from index infection. As a secondary outcome, we considered bloodstream reinfection only.

The effect of interest was the added risk of tolerant index infection vs nontolerant strain on outcome. We used survival analysis to account for early termination of follow-up because of death of patients (37% died within the 90 day follow-up). We used the Kaplan-Meier method to estimate the survival curves (ie, probability of not having a reinfection) within each group (tolerant vs nontolerant index infection). We estimated the average hazard ratio (HR) of tolerance and tested its significance using the Cox proportional-hazards regression model. To adjust for potential confounders, we applied the multivariate Cox regression model. We first included, in addition to tolerance, all covariates that were found significant (*P* < .05) in the univariate Cox regression and kept only significant covariates in the final model.

Because there were few cases of reinfection (22 for the primary outcome), we applied another approach as a sensitivity analysis: a Poisson regression model in which the dependent variable was the number of reinfections and the number of days in follow-up was used as an offset. Using the “exact” option enabled inference for small sample size [21]. This model is correct and has more power under the assumption that the risk for reinfection is constant during the follow-up.

The performance of the TDtest was carried out by comparing its results to the time-dependent killing assay, for 5 tolerant and 10 nontolerant isolates. Performance was expressed as sensitivity, specificity, and negative predictive value.

## RESULTS

### Incidence of BSI With Tolerant Strains and Reinfection Risk

In all, 16 285 blood cultures were obtained from November 16, 2017, through March 20, 2018, of which 1776 blood cultures were positive. Of positive samples, 204 (11.5%) grew *E. coli*, 100 of which were patient-unique (cohort 1). Three isolates were excluded from the analysis because they failed to grow, and an additional 3 because of mislabelling. Three additional patients were excluded from the final analysis because they died on the day of the index bacteremia.

**Figure 1.**
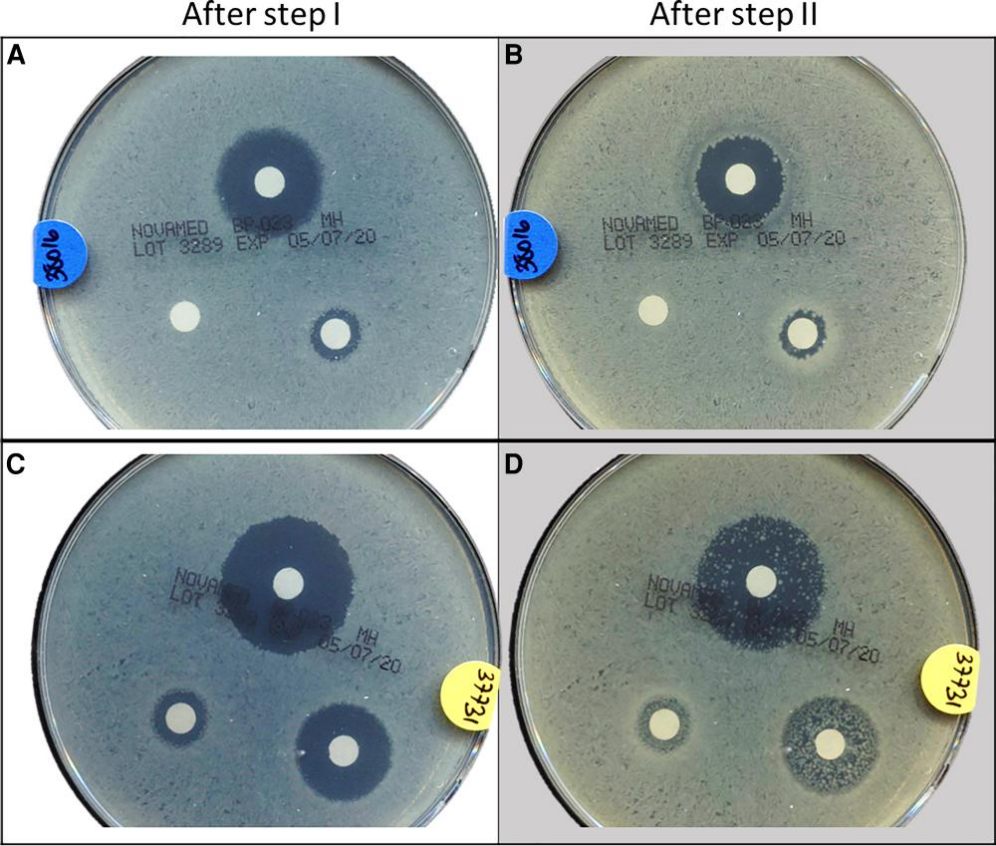
Representative TDtest results. Panels (*A*-*B*), negative TDtest; panels (*C*-*D*), positive TDtest. Two different strains from the prospective cohort were plated on Mueller Hinton plates with 3 Ertapenem discs (0.02, 0.2, and 2 µg). After the first incubation (*A*, *C*), 3 different inhibition zones appeared (small, medium, and large, respectively); the different strains show different radii corresponding to their different minimal inhibitory concentrations. Addition of nutrients to the discs and second incubation reveal differences in tolerance level. Although only 3 colonies grew of the nontolerant strain (*B*), hundreds of colonies of the other strain manifested a high level of tolerance (*D*). TDtest, Tolerance Disk Test.

Of blood isolates, 8 strains (8/94, 8.5%) were tolerant per the TDtest (Figure 1). The characteristics of cohort 1 patients are presented in Table 1. Patients with tolerant index infection had more previous episodes of infection, and more were diagnosed with a hematological disease.

**Table 1.** Baseline Characteristics of Patients With *E. coli* Bloodstream Infections (Cohort 1)

Covariate	Nontolerant Strain (n = 83)	Tolerant Strain (n = 8)	*P* Value
Age (mean ± SD), y	59.2 (±26.6)	55.5 (±29.9)	.526
Male	36.1%	75.0%	.054
Foreign body	27.7%	37.5%	.684
Past (90 d) infection	22.9%	62.5%	.028
Immunosuppression	9.6%	25.0%	.212
Hematological disease	7.2%	37.5%	.030
Diabetes mellitus	15.7%	12.5%	1.000
Any malignancy	26.5%	50.0%	.218
Solid tumor	20.5%	25.0%	.671
WBC, cells/mm^3^	12.3 (7.6)	9.8 (6.9)	.52
Neutropenia (ANC ≤500 PMN/mL^3^)	3.6%	12.5%	.312
Creatinine, mmol/L	99.3 (58.8)	104.3 (105.1)	.411
Albumin, g/L	30.4 (8.6)	27.9 (9.3)	.948
Urinary or biliary obstruction	32.5%	0%	.100
Abscess	8.4%	0%	1.00
Osteomyelitis	2.4%	0%	1.00
Hospitalization days (per 100 d follow-up)	3.1	11.5	<.001

Abbreviations: ANC, absolute neutrophil count; *E. coli*, *Escherichia coli*; PMN, polymorphonuclear; SD, standard deviation; WBC, white blood cell.

**Figure 2.**
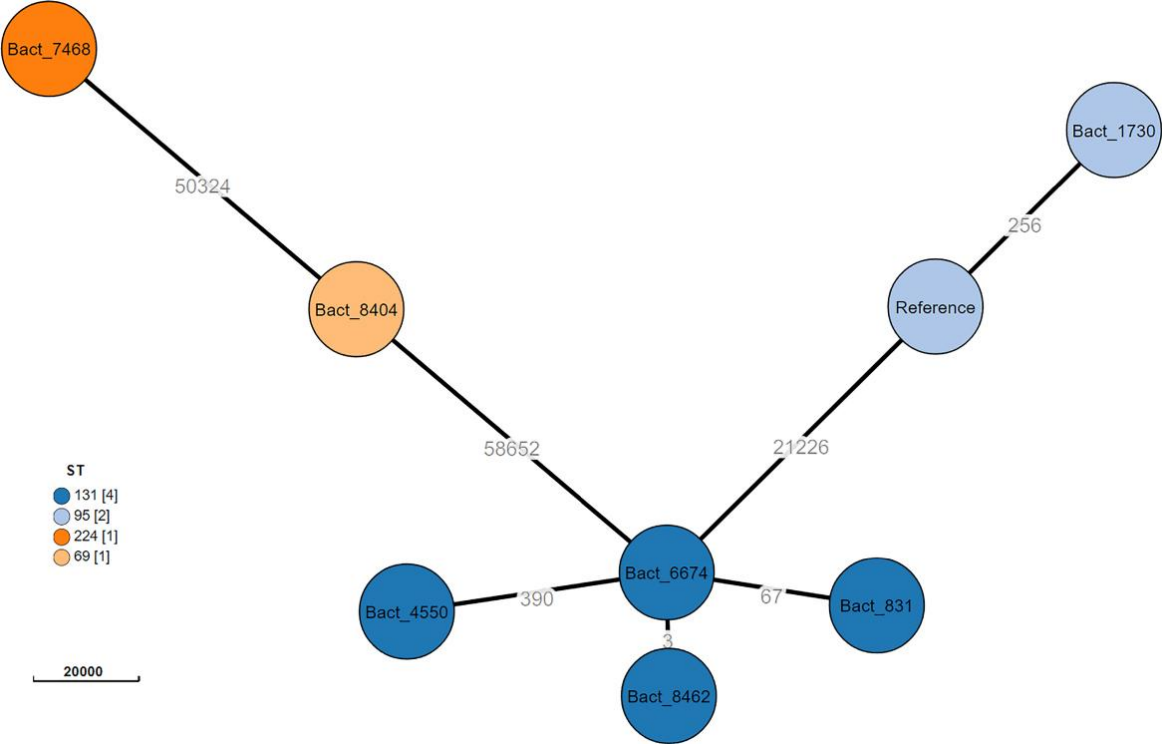
A phylogenetic analysis of index tolerant *Escherichia coli* isolates using core genome SNPs. The strain ExPEC XM was used as the reference. Each node represents a sequenced isolate and color coding denotes the assigned sequence type. The distances are the number of SNPs found between 2 samples (nodes). Abbreviation: SNP, single-nucleotide polymorphism.

Four of the 8 tolerant strains belonged to sequence type 131, which is known to be circulating in our hospital according to routine surveillance (unpublished data), and sequencing of 1 strain failed, whereas each of the remaining isolates were singletons. A phylogenetic analysis based on SNP mapping demonstrated that all isolates were unrelated, except for 2 of the ST131 isolates (Figure 2). One isolate was a carbapenemase producer (*bla*_KPC_).

**Figure 3.**
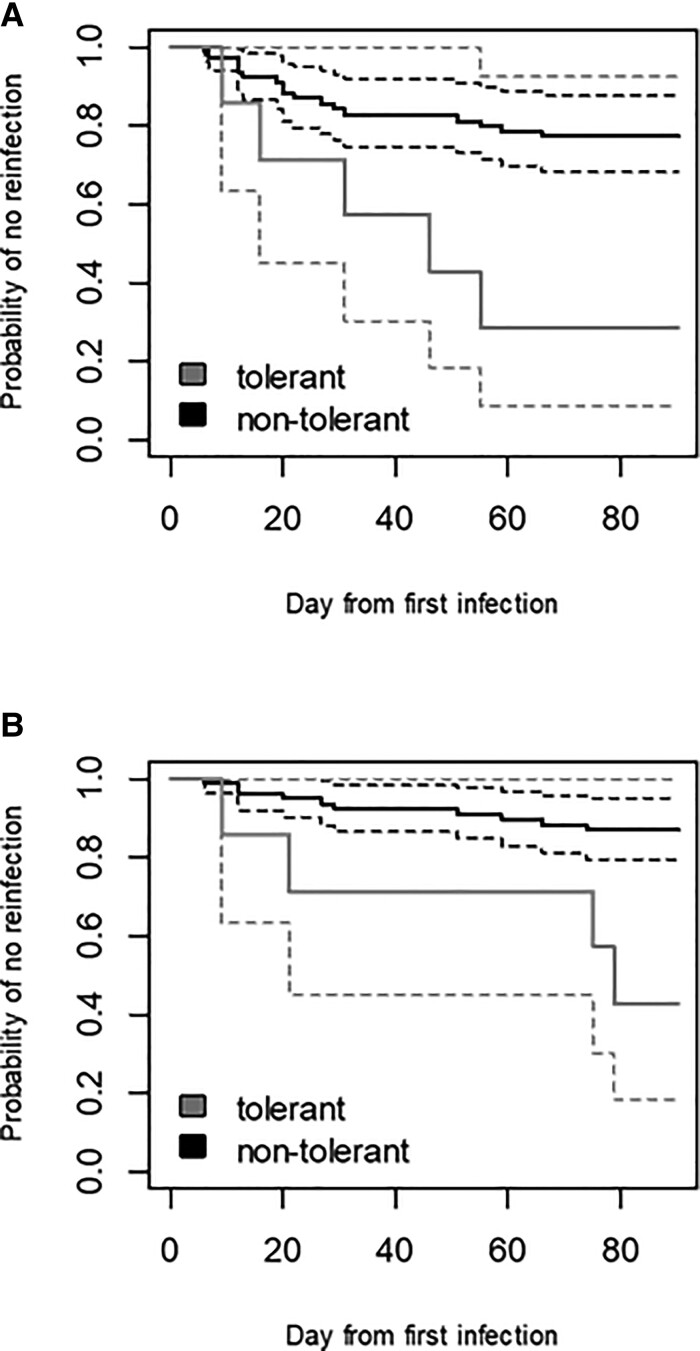
*A*, Survival curve for reinfection (BSIs or UTIs) among patients with index tolerant and nontolerant BSIs. *B*, Survival curve for reinfection (BSIs only) among patients with index tolerant and nontolerant bloodstream infections. The dashed lines are the 95% confidence intervals of the survival curve. Abbreviations: BSIs, bloodstream infections; UTIs, urinary tract infections.

The estimated survival curves of remaining noninfected for each group are shown in Figure 3*A*. The risk for reinfection in the patients infected by tolerant strains was significantly higher than those infected by nontolerant strains (HR, 4.1; 95% confidence interval [CI], 1.5–11.18; *P* [likelihood-ratio test] = .016).

In a univariate analysis, the following covariates were found to be significantly associated with reinfection: past infection, existence of an abscess, and number of days in hospital. In a multivariate Cox regression analysis, after adjusting for these covariates, the estimated risk for reinfection with an index tolerant strain remained high: HR, 3.98 (95% CI, 1.32–12.01) (Table 2).

**Table 2.** Multivariate Cox and Poisson Regression Analyses for Reinfection

Covariate	Hazard Ratio	95% Confidence Interval	*P* Value
**Cox regression analysis**			
Tolerance	3.98	(1.32–12.01)	.014
Past infection	2.53	(1.03–6.21)	.044
Abscess	4.89	(1.68–14.24)	.004
Hospitalization days	1.09	(1.04–1.14)	.001
**Poisson regression analysis**			
Tolerance	4.64	(1.55–13.87)	.025
Past infection	2.53	(1.04–6.13)	.040
Abscess	5.66	(1.96–16.32)	.001
Hospitalization days	1.08	(1.03–1.13)	.002

Applying the Poisson regression approach, patients with nontolerant strains were followed for 5502 patient days and had 17 reinfections, whereas the patients with index tolerant bloodstream isolates had 5 reinfections during 340 follow-up days. The estimated relative risk for reinfection with tolerant bacteria in a multivariate Poisson regression after controlling for the previous covariates was very similar at 4.64 (95% CI, 1.55–13.87), *P* = .025 using the “exact” method (Table 2).

For the secondary outcome, survival curves of the 2 groups are shown in Figure 3*B*. The estimated HR was 5.53 (95% CI, 1.7–17.98), *P* = .004. The relative risk of tolerance in the Poisson regression model was 5.86 (95% CI, 1.32–20.98), *P* = .021.

### Tolerant Strains Among the Recurrent *E. coli* Cohort

Because tolerance was found to be associated with an increased risk for additional infection within 90 days, we sought to explore the prevalence of tolerance among patients with established recurrent infection (cohort 2).

Between April 1, 2012, and April 30, 2017, there were 3929 patient-unique *E. coli* bloodstream episodes, including 32 patients with recurrent *E. coli* infections. For this analysis, we were able to retrieve 38 clinical isolates recovered from 21 patients. The characteristics of these patients are presented in Table 3.

**Table 3.** Baseline Characteristics of Patients with Paired Repeated *E. coli* Bloodstream Infections

Characteristics	TDtest positive	TDtest negative	*P* Value
Number	6	15	
Age (mean ± SD), y	65.4 ± 17	55 ± 12.6	.58
Female	3 (50%)	8 (53%)	.9
Hematological malignancy	4 (66%)	4 (26%)	.14
Other diseases^a^	2 (34%)	11 (73%)	.1
Time between BSI episodes (median), d	59	112	.51
Death (within 3 mo after the last positive culture)	4 (66%)	9 (60%)	1

Abbreviations: BSI, bloodstream infection; *E. coli*, *Escherichia coli*; SD, standard deviation; TDtest, Tolerance Disk Test.

a 4 cholangiocarcinoma, 3 cholelithiasis, and 1 each of drug-induced liver failure, cirrhosis, nephrolithiasis, pancreas carcinoma, primary sclerosing cholangitis.

The prevalence of tolerance among cohort 2 was higher than among the prospective cohort (cohort 1), 6/21 (28.6%) vs 8/94 (8.5) (*P* = .02), supporting the notion that tolerance is indeed associated with recurrent infection.

Hematological malignancy was more common among patients with tolerant strains; however, this difference was not statistically significant. Recurrence among patients with tolerant infection tended to occur earlier than those with nontolerant strains.

### Performance of TDtest Versus Kill Assay

Using a convenience sample of 15 isolates, 5 of which were TDtest negative, the sensitivity and specificity of the TDtest were 71.4% (95% CI, 29–96) and 85.7% (95% CI, 42–99), respectively. The negative predictive value was 96.9% (95% CI, 90–99).

### Performance of TDtest With Different Antimicrobials

Results of the TDtest with ampicillin, ceftriaxone, and ertapenem were concordant in 5 tolerant and 5 nontolerant isolates.

## DISCUSSION

This study sought to assess the incidence of tolerance among *E. coli* blood isolates and the risk for subsequent infection. Our study demonstrates that tolerant *E. coli* strains were not uncommon among bloodstream isolates and were associated with an increased risk of subsequent infection.

*E. coli* is the most common cause of bacteremia, and our findings are no exception. The epidemiology of BSIs in our hospital was typical to acute care hospitals: *E. coli* accounted for 11.4% of all bloodstream episodes, and the urinary tract was the main focus of infection, consisting of 41% of the infections [22, 23]. Of 15 *E. coli* strains that were typed, 5 belonged to ST131, which is also endemic in Israel [24].

*E. coli* BSIs are associated with frequent recurrence. There are several established risk factors for its recurrence: host-related factors include age, the severity of patient-related illnesses, hematological malignancy, structural abnormalities of the urinary or biliary tract, and related indwelling medical devices, whereas pathogen-related factors include uropathogenic *E. coli* and more recently ST131, which has been associated with recurrent UTI BSI episodes [23, 25–27].

Is tolerance an additional risk factor for recurrent infection? Tolerant *E. coli* can survive bactericidal antimicrobials [28] and become dominant under intermittent antibiotic exposure [13]. Tolerance in *E. coli* can be relatively easily induced in vitro [6, 10, 29]. There are, however, only limited data on the epidemiology of tolerance; late isolates of *Pseudomonas aeruginosa* from a patient with cystic fibrosis have been found to be fluoroquinolone-tolerant persister cells [4]. In addition, tolerance preceded resistance among isolates of *P aeruginosa* from a cohort of patients with cystic fibrosis [30]. More recently, 22.5% of randomly selected, genetically uncharacterized, uropathogenic *E. coli* strains isolated from community-acquired UTIs in Denmark were characterized as “quiescence,” a state proximal to tolerance [6]. Our findings corroborate these data and extend them. In the first study of a prospective cohort of *E. coli* BSIs, we found that 8.5% of the isolates were tolerant. Of note, except for 2 isolates that were closely related, tolerance was not a clonal phenomenon.

An additional important finding was that tolerance was an independent risk factor for subsequent infection. To validate this association, we looked for tolerance among a cohort of patients with recurrent infection. About one-third of the patients had bacteremia by a tolerant *E. coli*; moreover, reinfection among patients harboring a tolerant strain occurred sooner than among those with nontolerant strains.

The setup in which tolerance is of clinical significance is still unclear. Clinicians working in the inpatient setting are familiar with the high frequency of recurrence of urosepsis and biliary sepsis, especially among those with obstruction and the elderly. We hypothesized that the contribution of tolerance to repeated infections would be more evident among patients lacking any of these risk factors and those devoid of the natural protection provided by the immune system. Indeed, in the prospective cohort, hematological malignancy was found as a risk factor for reinfection, and among patients with recurrent infections, two-thirds of the patients with tolerant isolates suffered from a hematological malignancy vs one-quarter of those without. Nevertheless, this did not achieve statistical significance, likely because of the small sample size. Interestingly, in a recent report, tolerant *Staphylococcus aureus* were less common among neutropenic and steroid-treated patients [31]. Studying the collateral effect of specific chemotherapeutics on the selection of tolerance in bacteria is warranted. Tolerance is a resistance-independent phenomenon, and the in vitro evolution of resistance among tolerant strains is not by horizontal gene transfer. Thus, that one of the tolerant isolates was also carbapenemase-producing is worrisome.

A major impediment to the study of the epidemiology of tolerance to date has been the lack of an established laboratory assay [32]. We have recently reported on the TDtest, a simple modification of the agar disk diffusion method developed to identify tolerance [12]. Here, we successfully applied this assay in a clinical study. Moreover, we found that a negative TDtest strongly predicted that tolerance was not a potential risk factor for repeated infection. The TDtest is sensitive to growth conditions and has not yet been implemented in routine diagnostics [33]. We therefore corroborated the TDtest results with kill assays. Importantly, the negative predictive value of the TDtest was very high, suggesting that with a negative TDtest, other reasons for reinfection should be sought.

Our study has several limitations. Because in our screening we only tested tolerance to ertapenem, our findings regarding this phenotype are restricted to this drug. Nevertheless, we, and others, have shown concordant tolerance between different antimicrobials from the same class, such as ampicillin and ertapenem in *E. coli* [12], as well as from different classes, such as ofloxacin, carbenicillin, and tobramycin in *P aeruginosa* [4] and vancomycin and daptomycin but not gentamicin in *S aureus* [8, 34]. We have also observed similar findings in *E. coli*; tolerance, which is associated with growth impairment, typically implicates antimicrobials that are better at targeting growing bacteria such as all β-lactams, as well as fluoroquinolones. However, other antimicrobials such as kanamycin may still kill these tolerant bacteria. Therefore, the knowledge on the tolerance level of a strain may direct therapeutic choice [12]. It is plausible that a prediction of phenotype can be devised once the specific genetic mechanism is identified. This, however, was beyond the scope of this study.

This was an exploratory study, analyzing the association of tolerant *E. coli* BSIs with different outcomes. When analyzing many variables, there is a possibility that some results may be due to chance. Nevertheless, the association of tolerance with subsequent infection was as robust as to other known risk factors.

A limitation of our study was the lack of a pairwise comparison of samples. Thus, we were not able to perform analyses of the molecular mechanisms that underlie these events. Analyses of consecutive isolates of *Enterococcus faecium* bacteremia that persisted despite appropriate antibiotic therapy identified the emergence of a single mutation associated with tolerance [35]. In addition, monitoring *S aureus* strains evolving in patients under treatment enabled the detection of emerging tolerant mutants, followed by the emergence of resistance [8]. Recurrent UTIs are frequently caused by the same *E. coli* strain [27]. Because our repository only spans consecutive bloodstream isolates and isolates from proximal bacteremia episodes were not kept, we were not able to demonstrate that the tolerant *E. coli* was directly responsible for the subsequent infection.

Finally, this was a single-center study and thus the overall number of tolerant bacteria included was small. Additional studies, enrolling patients with various types of infections, across various centers, and analysis of a larger database of tolerant isolates are warranted.

In conclusion, we show here that tolerance, as determined by TDtest, is a novel risk factor for reinfections. Further studies on other bacterial populations and patients are needed to corroborate our findings. We demonstrate that the TDtest is practicable for detection of tolerance and implementing this method could strengthen the detection of tolerance and improve future patient management. The genomic mechanisms underlying tolerance should be thoroughly investigated to inform future surveillance efforts.

## Notes

***Financial support.*** This work was supported by the Minerva Foundation, the Israel Science Foundation (grant No. 597/20), and by the Kamin program of the Israel Innovation Authority (75090) to N.Q.B.
